# Incidence and clinical impact of renal failure and bleeding following transcatheter tricuspid valve annuloplasty

**DOI:** 10.1007/s00392-024-02388-4

**Published:** 2024-02-15

**Authors:** Thorsten Gietzen, Jan Althoff, Laurin Ochs, Muhammed Gerçek, Jennifer von Stein, Caroline Hasse, Christos Iliadis, Kai Friedrichs, Volker Rudolph, Stephan Baldus, Roman Pfister, Maria Isabel Körber

**Affiliations:** 1 Faculty of Medicine and University Hospital Cologne, Clinic III for Internal Medicine, University of Cologne, Kerpener Straße 62, 50937 Cologne, Germany; 2 Clinic of General and Interventional Cardiology/Angiology, Heart and Diabetes Center Northrhine-Westfalia, Ruhr University Bochum, Bad Oeynhausen, Germany

**Keywords:** Tricuspid disease, TTVR, Bleeding, Renal insufficiency

## Abstract

**Background:**

Bleeding is the most common complication after percutaneous leaflet-based tricuspid valve repair and associated with acute kidney injury (AKI) and adverse outcome. TTVA with the Cardioband system is a technically more complex procedure; however, frequency and prognostic impact of postinterventional bleeding and renal complications have not been thoroughly examined.

**Aims:**

This study was performed to determine the incidence and clinical impact of bleeding complications (MVARC criteria) and acute kidney injury (KDIGO criteria) following transcatheter tricuspid valve annuloplasty (TTVA).

**Methods:**

In a bi-center retrospective analysis of patients undergoing TTVA between 2018 and 2022, we examined frequency, predictors, and clinical impact of bleeding and renal failure.

**Results:**

In 145 consecutive patients, the incidence of any MVARC bleeding was 20.7% (*n* = 30), whereas major MVARC bleeding occurred in 6.9% (*n* = 10). The incidence of AKI was 18.6% (*n* = 27). Risk factors for bleeding events included low baseline hemoglobin and elevated baseline creatinine levels. Risk factors for AKI included diabetes mellitus, arterial hypertension, high body mass index, and elevated baseline creatinine levels. Neither procedure duration nor amount of contrast media was associated with AKI or bleeding. Both bleeding and AKI led to a longer hospital stay.

At 3 months, 10.0% (*n* = 3) of patients with bleeding and 7.8% (*n* = 9) of patients without bleeding complications died (*p* = 0.70). Additionally, mortality rate was 7.4% (*n* = 2) in patients with AKI compared to 8.5% (*n* = 10) without AKI (*p* = 0.83).

**Conclusion:**

While about a fifth of patients undergoing TTVA suffered from postinterventional AKI or bleeding, none of these complications was associated with higher mortality at short-term follow-up. One important risk factor for both complications was chronic renal dysfunction, indicating a high-risk patient population. The most frequent bleeding localizations were the femoral access site, pericardial hemorrhage, and the esophagus, which need explicit attention in periprocedural management.

**Graphical Abstract:**

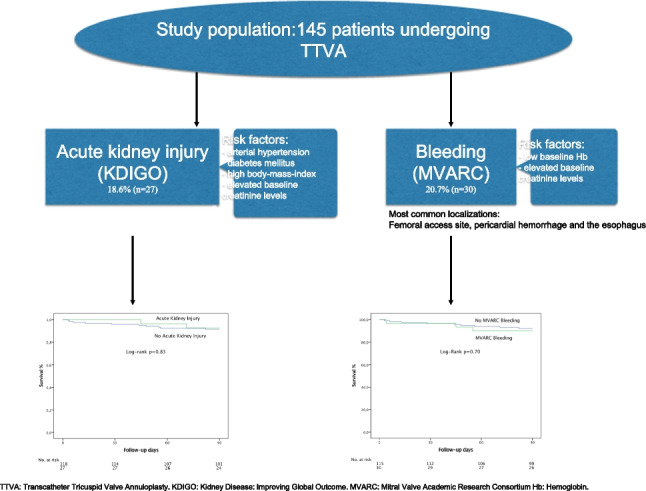

## Introduction

Clinically relevant tricuspid regurgitation (TR) is a common entity especially in elderly patients. A prevalence of 4% in people aged 75 years or older was described and TR is associated with excess mortality on the community level comprising an important public health problem [1, 2].

Until recently, therapeutic options for the correction of TR were scarce. Due to various comorbidities and the corresponding high periprocedural risk, less than 3% of patients with isolated moderate-to-severe TR undergo corrective surgery [2, 3]. Recently, catheter-based therapies emerged as efficacious in improving TR with a low periprocedural risk in preliminary observational studies [4–6]. CE-approved transcatheter repair techniques for TR include leaflet approximation and direct annuloplasty with the Cardioband™ system [7, 8]. The patient collective is mostly very frail and there is no evidence yet showing a mortality benefit, which makes periinterventional safety a major concern. Periinterventional bleeding and acute kidney injury have been shown to be relevant complications after percutaneous aortic and mitral valve replacement or repair [9–11]. Observations made in the context of transcatheter mitral valve repair showed an incidence of MVARC bleeding of 21.6% and acute kidney injury of 17.9%. Additionally, acute kidney injury has been associated with higher mortality in surgical and transcatheter mitral valve repair [10, 12].

Data specifying the frequency and relevance of complications in percutaneous tricuspid valve repair via direct annuloplasty (TTVA) are still lacking. Direct annuloplasty is a technically more complex procedure compared to leaflet-based interventions, with regard to procedure time and the need for arterial access and contrast media to visualize the right coronary artery. Yet, it is unclear whether this imposes a significant risk for bleeding or kidney injury and whether these complications may impact clinical outcome.

Therefore, this study was performed to determine incidence, predictors, and impact of bleeding complications and acute kidney injury following direct TTVA.

## Materials and methods

### Study population

Performing a bi-center retrospective analysis including all consecutive patients undergoing TTVA between 2018 and 2022 at two German high-volume institutions (Heart Center of the University Hospital of Cologne and Heart and Diabetes Center Bad Oeynhausen), we retrospectively analyzed the incidence and impact of bleeding complications and acute kidney injury. In total, 145 consecutive patients were included in the final analysis. Of these, 81 patients were treated at Cologne Heart Center, 64 at Bad Oeynhausen Heart Center.

All patients were discussed in an interdisciplinary heart team and deemed inoperable. The individual decision to perform TTVA was made considering anatomical features that made them less suitable for leaflet-based therapy (considering—among other factors—leaflet morphology, severity of coaptation gap, annular size, and leaflet tethering). Further recommendations on patient selection were made by the device manufacturer after evaluation of transthoracic and transesophageal echocardiography, cardiac computed tomography, and coronary anatomy.

### Data assessment

Data were obtained using the hospital’s internal digitalized patient files. Detailed procedural reports, as well as procedural angiography and echocardiography, formed the basis of the data on intraprocedural complications. Follow-up and mortality data were retrieved during routine visits to our outpatient clinic. Where this was not possible, patients or their general practitioners were contacted by phone. Survival data were regularly obtained from the German Civil Registry or via routine follow-up visits. Data assessment was approved and overseen by the local ethics committees of the University of Cologne and Ruhr-University Bochum, respectively. All data collection was performed within a prospective local register, and patients gave informed consent. Central analysis was based on anonymized data.

Severity of TR was assessed using transthoracic echocardiography. In accordance with current recommendations, vena contracta diameter (VC), regurgitant volume estimated with the proximal isovelocity surface area (PISA), and effective regurgitant orifice area (EROA) were recorded for assessing TR severity. [13, 14] TR was graduated into five grades according to classification of Hahn et al. as follows: trace (0), mild (I), moderate (II), severe (III), massive (IV), and torrential (V) [15].

Additionally, clinical assessment, basic physical examination, and, if necessary, laboratory tests, were performed.

### Procedure

Electrocardiogram-triggered full-cycle cardiac computed tomography scan was used to plan the procedure (planning views, implant length, and evaluation of proximity to right coronary artery and coronary sinus). During the TTVA procedure, coronary angiography is performed through an arterial femoral access (6 French) and a guide wire is inserted as a landmark for the implantation and to assess potential right coronary artery (RCA) damage.

The procedure is guided mostly through transesophageal echocardiography and also fluoroscopy. The device is inserted using femoral access (26-French venous access). The Cardioband system itself comprised a Dacron band enclosing a wire that is secured to the tricuspid annulus through multiple anchors (stainless steel screws), reaching from 12 (size A) to 17 (size F). Following fixation, the annulus is contracted using a cinching handle.

During the procedure, unfractionated heparin is administered with a target activated clotting time of 250 to 350 s. At the end of the procedure, venous access site is closed using a Z-suture and the arterial access site is closed using a vascular closing device (Angio-Seal®, 6 French, Terumo). In patients receiving oral anticoagulants, the drug is usually continued on the first postinterventional day.

After intervention, patients were generally moved to the intensive care unit (ICU) for at least 24 h, where monitoring involved ECG, lab tests, and echocardiographic evaluations to detect potential pericardial effusions.

### Endpoints

We adopted the criteria outlined by the Mitral Valve Academic Research Consortium (MVARC) to identify bleeding complications. [16] Here, minor bleeding is defined as any overt bleeding requiring nonsurgical medical intervention by a health care professional or increased level of care, prompting evaluation or requires one or two red blood cell (RBC) transfusions and otherwise does not meet criteria for major, extensive or life-threatening bleeding. Major bleeding is defined as an overt bleeding associated with a drop in hemoglobin of ≥ 3.0 g/dl and extensive MVARC bleeding as an overt bleeding with hemoglobin drop of ≥ 4 g/dl, respectively.

Life-threatening bleeding is defined as bleeding in a critical organ such as intracranial, intraspinal, intraocular, or pericardial necessitating surgery or intervention or causing hypovolemic shock or hypotension (systolic blood pressure < 90 mmHg).

Fatal bleeding is defined as bleeding adjudicated as being a proximate cause of death. Severe bleeding adjudicated as being a major contributing cause of a subsequent fatal complication, such as myocardial infarction or cardiac arrest, is also considered fatal bleeding.

Baseline hemoglobin level was determined using the most recent serum hemoglobin, mostly taken on admission to hospital, and hemoglobin nadir was defined as the lowest value during the postinterventional hospital stay. Due to the retrospective character of the study, hemoglobin levels were not evaluated in a standardized manner. In general, hemoglobin levels were assessed at least once daily during intensive care unit stay and at the physicians’ discretion thereafter.

Acute kidney injury (AKI) was defined according to the Kidney Disease Improving Global Guidelines (KDIGO). [17].

KDIGO criteria define AKI as a maximal change in serum creatinine from baseline and is divided in three stages:

I. Increase in serum creatinine by ≥ 0.3 mg/dl (≥ 26.5 mol/l) within 48 h, an increase in serum creatinine to ≥ 1.5 times baseline within the previous 7 days or urine volume ≤ 0.5 ml/kg/h for 6 h
II. Increase in serum creatinine > 2.0- to 3.0-fold from baseline or urine output < 0.5 ml/kg/h for > 12 h but < 24 h

III. Increase in serum creatinine < threefold or increase of serum creatinine ≥ 4.0 mg/dl (≥ 354 μmol/l) with an acute increase of at least 0.5 mg/dl (44 μmol/l) or urine output < 0.3 ml/kg/h for > 24 h, or anuria for > 12 h

Patients receiving renal replacement therapy are considered stage 3 irrespective of other criteria.

Baseline renal function was determined by using the most recent serum creatinine, mostly taken on admission to hospital. Due to the retrospective character of the study, creatinine levels were not evaluated in a standardized manner. In general, laboratory evaluation was conducted at least once daily during intensive care unit stay and at the physicians’ discretion thereafter. Urine output was not measured routinely and only monitored in selected patients with impaired kidney function and signs for impairment.

### Statistical analysis

Data were collected and statistical analysis performed using IBM SPSS® Version 23.0.

Descriptive data are presented by relative and absolute frequencies. Median, as well as arithmetic mean (if applicable), and standard deviation are given. The Shapiro–Wilk test was utilized to assess the normal distribution of the variables. To verify the distribution of non-parametric variables, the Mann–Whitney *U* test was used. For the statistical comparison of categorical variables, the Pearson chi-square test was chosen whenever possible. In cases where this was not possible, the Fisher exact test was used. Survival data is presented in the form of Kaplan–Meier curves; survival testing was performed using the log rank test.

The two-tailed significance level of *p* = 0.05 was chosen.

## Results

### Baseline demographics

Patients presented with a median age of 79 (74–82) years. The median EuroScore II was 4.16% (2.83–7.33). Over a third of patients suffered from torrential tricuspid valve regurgitation (39.6%) resulting in dyspnea mostly grade III according to NYHA functional class (84.1%). On average, patients presented with mildly impaired renal function and anemic at baseline (median Hb 12.0 g/dl, IQR 10.8–13.3) and creatinine level 1.20 mg/dl, IQR (0.87–1.63). Median TRI-SCORE was 5 IQR [3–6].

Baseline demographics are presented in Table 1.

**Table 1:** Baseline characteristics

Event	All patients *n* = 145	No MVARC bleeding	MVARC bleeding	*P*-value *Comparison MVARC/no MVARC bleeding	No AKI	AKI	*P*-value *comparison AKI/no AKI
Female (%)	107 (73.8)	85 (73.9)	22 (73.3)	0.95	87 (73.7)	20 (74.1)	0.97
Age median (IQR)	79 (74–82)	79 (74–81)	80 (75–82)	0.36	80 (74–82)	78 (71–81)	0.19
BMI mean ± SD	26.8 ± 5.4	26.0 (22.9–29.4)	26.5 (24.0–28.2)	0.35	25.5 (23.1–29.4)	28.7 (22.8–33.5)	**0.03**
Atrial fibrillation	130 (89.7)	102 (88.7)	28 (93.3)	0.83	106 (89.8)	24 (88.9)	0.51
Arterial hypertension	100 (69.0)	76 (66.1)	24 (80.0)	0.14	86 (72.9)	14 (51.9)	**0.03**
Diabetes mellitus	38 (26.2)	26 (22.6)	12 (40.0)	0.54	26 (22.0)	12 (44.4)	**0.02**
Hb baseline median (IQR)	12.0 (10.8–13.3)	12.2 (11.0–13.4)	10.9 (9.1–12.2)	** < 0.01**	12.1 (10.9–13.3)	11.7 (10.3–13.2)	0.36
GFR baseline median (IQR)	48 (35–65)	53 (35–68)	39 (30–56)	**0.02**	53 (34–67)	39 (35–53)	**0.02**
Creatinine (mg/dl) (IQR)	1.20 (0.87–1.63)	1.10 (0.85–1.59)	1.38 (0.95–1.89)	**0.03**	1.10 (0.86–1.50)	1.47 (3.17–7.28)	**0.04**
Advanced chronic kidney disease (estimated GFR < 45 ml/min/m^2^)	68 (46.9)	49 (42.6)	19 (63.3)	**0.04**	49 (41.5)	19 (70.4)	** < 0.01**
Dialysis (%)	9 (6.2)	6 (5.2)	3 (10.0)	0.39	8 (6.8)	1 (3.7)	1.00
NYHA functional class							
I	0 (0)	0 (0.0)	0 (0.0)	1.00	0 (0.0)	0 (0.0)	1.00
II	11 (7.6)	8 (7.0)	3 (10.0)	0.70	9 (7.6)	2 (7.4)	1.00
III	122 (84.1)	99 (86.1)	23 (76.7)	0.21	100 (84.7)	22 (81.5)	0.77
IV	12 (8.3)	8 (7.0)	4 (13.3)	0.26	9 (7.6)	3 (11.1)	0.70
Grade of tricuspid regurgitation							
Severe	47 (32.4)	38 (33.0)	9 (30.0)	0.75	39 (33.1)	8 (29.6)	0.73
Massive	41 (28.3)	36 (31.3)	5 (16.7)	0.17	37 (31.4)	4 (14.8)	0.10
Torrential	57 (39.6)	41 (36.0)	16 (53.3)	0.08	42 (35.9)	15 (55.6)	0.06
Euroscore median (IQR)	4.16 (2.83–7.33)	4.03 (2.68–7.46)	4.59 (2.95–7.00)	0.79	3.89 (2.50–7.33)	5.11 (3.17–7.28)	0.15
Tri-Score median (IQR)	5 (3–6)	4 (3–6)	6 (4–6)	0.06	4 (3–6)	6 (4–7)	0.10
LVEF (%) mean ± SD	55 ± 9	56 ± 9	58 ± 8	0.06	55 ± 9	53 ± 10	0.10
Oral anticoagulants	135 (93.1)	107 (93.0)	28 (93.3)	1.00	110 (93.2)	25 (92.6)	1.00

Data presented in %

*BMI*, body mass index; *NYHA*, New York Heart Association; *AKI*, acute kidney injury; *IQR*, interquartile range; *SD*, standard deviation; *Hb*, hemoglobin; *GFR*, glomerular filtration rate; *LVEF*, left ventricular ejection fraction

Baseline characteristics of patients undergoing transcatheter tricuspid valve annuloplasty (*TTVA*)

### Procedure

Patients were hospitalized for a median of 8 days (6–10 days) and spent a median of 2 days (1–4 days) on ICU. Median procedure time was 198 min (166–238 min), with a median contrast agent usage of 89 ml (65–139 ml). Technical success, defined as patient being alive, device deployed, and no emergency intervention needed, was achieved in 97.9% (142/145 patients). One patient required emergency heart surgery for intramural myocardial hematoma following primary covered stent intervention after RCA perforation and cardiac tamponade. This patient had a 35-day hospital stay and survived for 580 days (with no cardiovascular cause of death). In another case, the TTVA procedure was terminated prematurely due to an unfavorable vena cava opening angle that made device steering impossible. Additionally, one intervention failed because first anchor implantation was impossible, leading to procedure termination and subsequent surgical valve operation.

Four patients died during hospital stay (2.8%). One patient died of sudden cardiac death 3 days after the intervention, while he was already on the standard care ward. The postmortem pacemaker exam showed a ventricular tachycardia as the probable cause of death. One patient with history of CABG surgery experienced new-onset dyspnea but no angina after TTVA. A repeat coronary angiography showed no relevant new coronary stenosis. The patient died on postinterventional day 5 after excluding reversible causes and following 60 min of cardiopulmonary resuscitation due to pulseless electrical activity, with an eventually unclear cause of death. Further causes of death included pneumonia due to aspiration after stroke (*n* = 1) and one patient died following septic shock after the development of ventilator-associated pneumonia and enteritis leading to toxic megacolon.

### Bleeding

The overall incidence of any MVARC bleeding was 20.7% (*n* = 30), whereas major MVARC bleeding (hemoglobin drop ≥ 3 g/dl) occurred in 6.9% (*n* = 10). A hemoglobin drop of ≥ 4 g/dl occurred in eight cases (5.5%). Four patients suffered from a life-threatening bleeding complication (2.8%). In eight (5.5%) patients, minor bleeding (overt bleeding, but hemoglobin drop < 3 g/dl) was detected. Transfusion of packed red blood cells was needed in 16 of all procedures (11.0%), and 15 patients (10.3%) required an intervention (pericardiocentesis, ösophagogastroduodenoscopy, surgery) to stop bleeding. Bleeding complications are listed in Table 2.

**Table 2:** Bleeding complications

Event	Patients *n* = 145
MVARC bleeding	30 (20.7)
Minor bleeding	8 (5.5)
Major bleeding	10 (6.9)
Extensive bleeding	8 (5.5)
Life-threatening bleeding	4 (2.4)
Lethal bleeding	0 (0.0)
Bleeding requiring intervention	15 (10.3)
Transfusion of red blood cells	16 (11.0)

Data presented in %

Bleeding complications according to the MVARC definition: minor bleeding (overt bleeding < 3 g/dl), major bleeding (> 3 g/dl), extensive bleeding (> 4 g/dl)

*MVARC*, Mitral Valve Academic Research Consortium

Patients who suffered from bleeding complications presented with a significantly lower Hb (10.9 vs. 12.2 g/dl; *p* < 0.01) and higher creatinine levels (1.38 vs. 1.10 mg/dl; *p* = 0.03), and showed a higher prevalence of advanced chronic kidney disease (GFR < 45 ml/m^2^) (63.3% vs. 42.6%; *p* = 0.04) at baseline.

The presence of periinterventional AKI was not significantly associated with the occurrence of bleeding complications (*p* = 0.13).

**Fig. 1:**
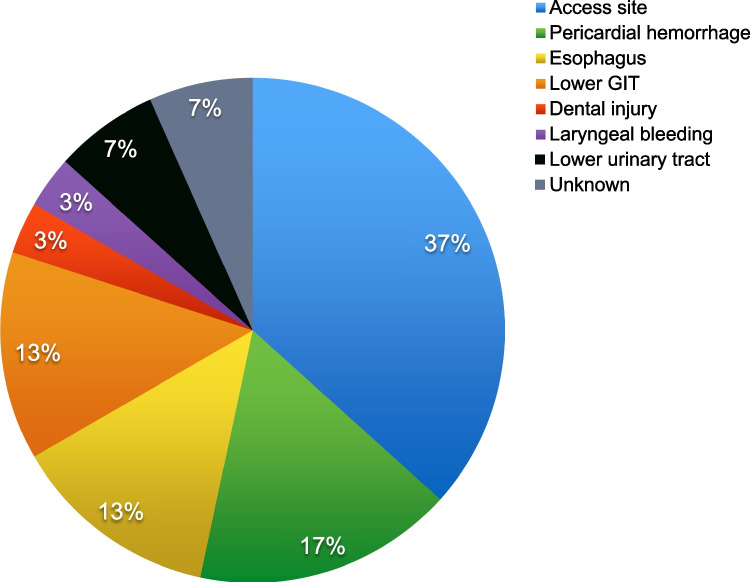
Bleeding localization. Bleeding locations after direct annuloplasty: most common locations were access site bleeding (37.7%), followed by pericardial hemorrhage and esophageal bleeding

Most commonly, periinterventional bleeding was related to the femoral access site (*n* = 11; 37%) followed by pericardial hemorrhage (*n* = 5; 17%), esophageal bleedings (*n* = 4; 13%), and bleeding of the lower gastrointestinal tract (*n* = 4; 13%). Other bleeding complications occurred in the lower urinary tract (*n* = 2) and the laryngeal area (*n* = 1), and due to dental injury (*n* = 1). A drop in hemoglobin levels ≥ 3 g/dl without a clinically visible bleeding sign—not considered in the MVARC bleeding definition—occurred in 7% of patients (*n* = 2). Bleeding localizations are shown in Fig. 1.

Patients who suffered a bleeding complication had a longer stay on ICU (4 vs. 2 days, *p* < 0.01) as well as a longer overall hospitalization (10 vs. 7 days, *p* < 0.01).

**Fig. 2:**
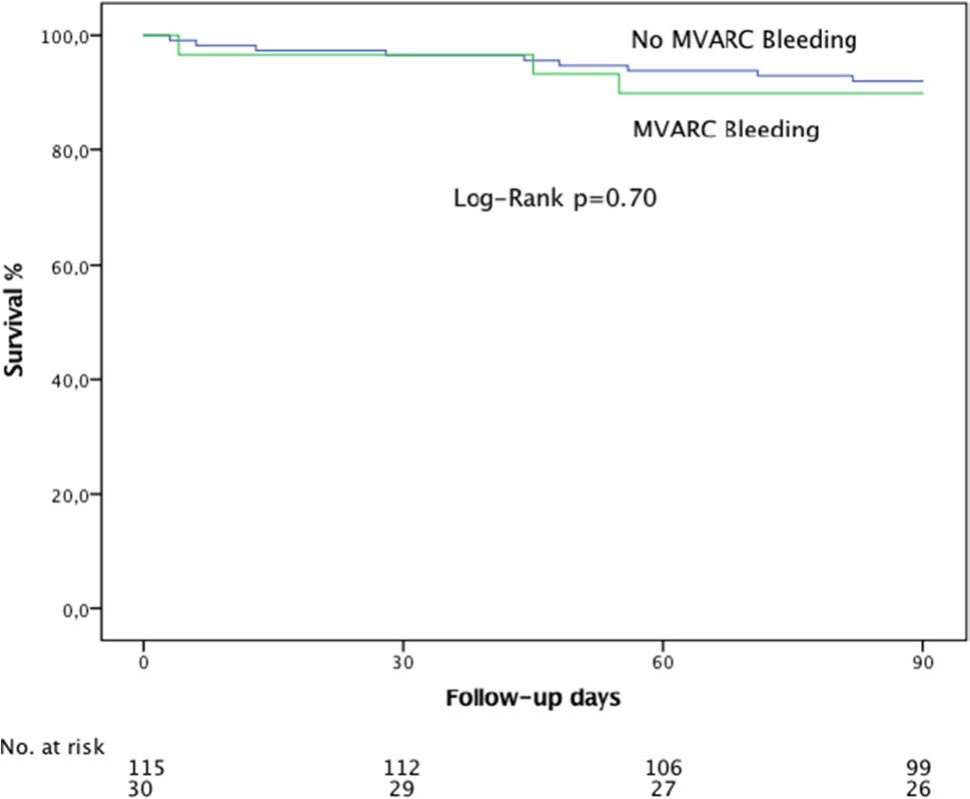
Overall survival of patients with and without MVARC bleeding. Survival of patients treated with direct annuloplasty was examined with Kaplan–Meier analysis. There was no significant difference in survival comparing patients with and without bleeding complications during follow-up of 90 days (*p* = 0.70)

There was no bleeding being a proximate cause of death in all 145 patients who underwent TTVA. The presence of an MVARC bleeding was not associated with a significantly increased overall mortality during short-term follow-up of 90 days (10.0% patients with bleeding vs. 7.8% patients without bleeding, *p* = 0.70, Fig. 2).

### Acute kidney injury

The overall incidence of MVARC AKI was 18.6% (*n* = 27) with a majority of patients suffering from stage 1 AKI (13.8%, *n* = 20).

A stage 2 AKI occurred in 1.4% (*n* = 2) and stage 3 in 3.4% of patients (*n* = 5). There were two patients (1.4%) with postinterventional need for new renal replacement therapy (Table 3).

**Table 3:** Acute kidney injury

Event	Patients *n* = 145
Acute kidney injury	27 (18.6)
- Stage 1	20 (13.8)
- Stage 2	2 (1.4)
- Stage 3	5 (3.4)
New renal replacement therapy	2 (1.4)

Data presented in %. Acute kidney injury according to the *KDIGO* (Kidney Disease Improving Global Outcomes) definition

Median creatinine level was 1.20 mg/dl (0.87–1.63 mg/dl) at baseline, 1.24 mg/dl (0.83– 0.80 mg/dl) at 7 days after the procedure, and 1.20 mg/dl (0.92–1.69 mg/dl) at 3 months follow-up.

The incidence of diabetes mellitus (23.6% vs. 45.0%; *p* < 0.027) and arterial hypertension (57.7% vs. 77.7%; *p* = 0.04) was higher in patients who developed acute kidney injury. Furthermore, patients suffering from AKI had a higher BMI (28.7 vs. 25.5; *p* = 0.03) and presented initially with elevated levels of serum creatinine (1.47 mg/dl vs. 1.10 mg/dl; *p* = 0.04).

The presence of MVARC bleeding was not associated significantly with AKI (*p* = 0.11).

Neither amount of contrast media (*p* = 0.31) nor procedural duration (*p* = 0.75) correlated with the development of AKI.

Minimal periprocedural mean arterial pressure in patients with AKI varied between 49 and 112 mmHg (median 65 mmHg). In those patients without AKI mean arterial pressure that varied between 39 and 83 mmHg (median 63 mmHg). There was no statistically significant difference between groups (*p* = 0.45).

Patients who developed an acute kidney injury had a longer overall hospitalization (9 vs. 7 days, *p* < 0.01). Additionally, these patients had a longer stay on ICU (4 vs. 2 days, *p* = 0.055) which was not statistically significant.

**Fig. 3:**
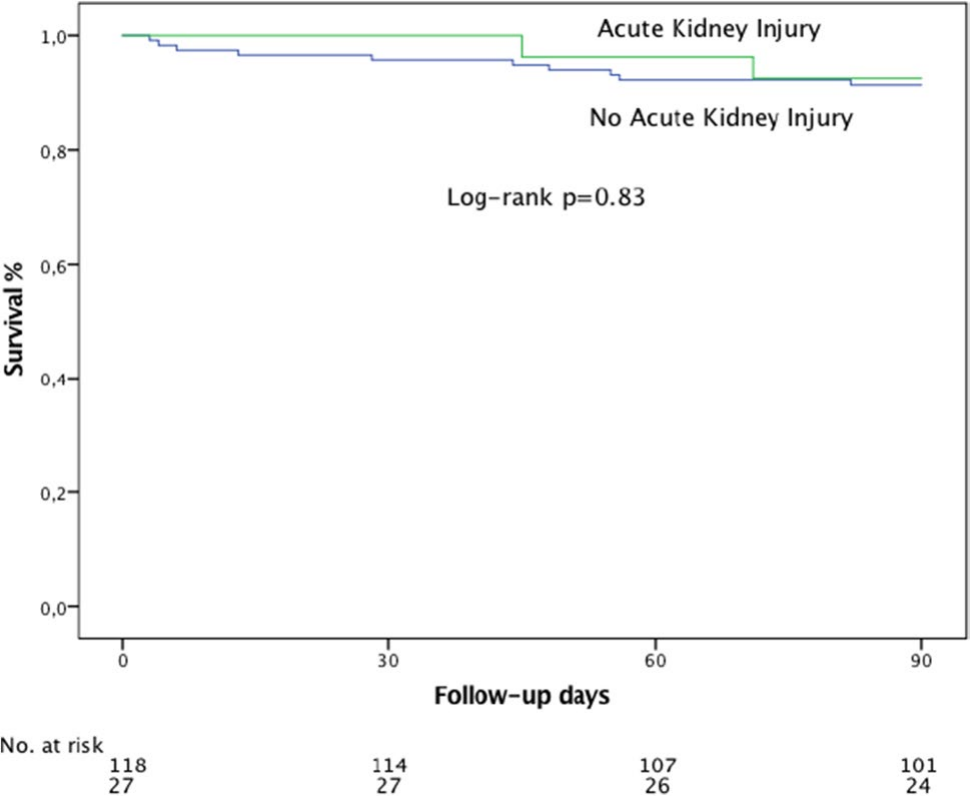
Overall survival of patients with and without acute kidney injury. Survival of patients treated with direct annuloplasty was examined with Kaplan–Meier analysis. There was no significant difference in survival comparing patients with and without acute kidney injury during follow-up of 90 days (*p* = 0.83)

The presence of MVARC acute kidney injury was not associated with a significantly increased overall mortality during short-term follow-up of 90 days (7.4% patients with AKI vs. 8.5% patients without AKI, *p* = 0.83, Fig. 3).

## Discussion

To the best of our knowledge this is currently the first study to determine incidence, predictors, and impact of bleeding complications and acute kidney injury following TTVA in a large, multi-center real-world cohort.

We demonstrated as follows:

(I) About a fifth of patients suffered bleeding complications or acute kidney injury following TTVA
(II) In this study population, none of these complications was associated with higher mortality rates at short-term follow-up, but
(III) Bleeding as well as acute kidney injury led to a longer ICU and overall hospital stay
(IV) Femoral access site, pericardial hemorrhage, and the esophagus were the most common bleeding localizations
(V) While chronic kidney disease and anemia at baseline were predictive of bleeding, neither amount of contrast media nor procedure duration was associated with bleeding or AKI following TTVA

Transcatheter tricuspid valve repair is an emerging treatment option for relevant tricuspid regurgitation (TR) in high-risk patients with an excellent safety profile in preliminary observational studies [4–6]. Within catheter-based techniques, leaflet-based repair is the most widely used technique and here periinterventional bleeding complications have been reported in less than 10% of patients [4, 5].

In the recently published TRILUMINATE Pivotal trial, nine out of 175 patients (5.2%) undergoing tricuspid edge-to-edge repair (TEER) suffered from periinterventional major bleeding [18]. A direct comparison between TTVA and TEER, performed by our study group, suggests that bleeding complications are more than twice as frequent after TTVA treatment as after TEER. The study included 74 patients treated with TTVA. Here, major or more severe bleeding occurred in 9.2% of TEER and 20.3% of TTVA patients (*p* = 0.049) without any fatal bleedings [19].

These data may lead to the assumption that bleeding is more frequent after TTVA than after leaflet-based repair. One potential explanation might be the additional need for arterial access to visualize the right coronary artery. In our study, arterial access was established using only a small femoral sheath (6F) and sealed with a closure device (Angio-Seal®, Terumo) after the procedure. Further significant bleedings were localized at the esophagus that were mostly related to periprocedural imaging with TEE.

When comparing the earliest with later procedures, it is of note that three out of four esophageal bleedings occurred within the first 20 procedures performed. This highlights the importance of operator experience, with TTVA being a complex procedure for the interventional echocardiographer.

Additionally, patients with right-sided heart failure may present in general with a higher rate of esophageal varices due to congestion. Furthermore, it has to be noted that in abovementioned prospective clinical studies on patients undergoing leaflet-based therapy, these were highly selected, and bleeding was not defined as any MVARC bleeding, but as major (hemoglobin drop > 3 g/dl) or more extensive bleeding. This might limit the comparability of the different collectives.

TTVA procedures pose the risk of unfavorably long intervention duration, so this might per se act as a risk factor for bleeding complications due to prolonged exposure to heparin. It has been previously reported that bleeding complications after transcatheter mitral valve repair are associated with longer procedural duration [11]. However, in our study, procedure duration was not associated with bleeding.

Another known risk factor for bleeding in catheter-based valve therapy is advanced kidney disease [11]. We could support this finding in our cohort, as patients with bleeding suffered more often from impaired kidney function at baseline. Furthermore, low baseline hemoglobin values were independently associated with bleeding complications and anemia is a known risk factor for bleeding complications [9, 11]. While chronic kidney disease and anemia are common comorbidities characterizing an elderly and frail cohort, it seems imperative to pay close attention to periprocedural safety in these patients. In daily practice, this might include considerations of a radial instead of femoral arterial access, especially careful TEE guiding to avoid mucosal trauma as well as use of novel imaging techniques such as intracardiac echocardiography or 3D imaging and multiplanar reconstruction to avoid frequent changes from transesophageal to transgastric windows.

While bleeding affected a fifth of patients and was associated with a longer ICU and overall hospital stay, there has been no fatal bleeding event in the observed 145 patients and there was no significant association of bleeding with mortality at short-term follow-up, as has been already suggested in previous studies on patients undergoing transcatheter mitral repair or aortic replacement [9, 11].

While bleeding is one of the most common and feared complications after transcatheter therapies, AKI has been also described frequently and with negative impact on prognosis after aortic and mitral valve interventions [10, 11]. Most interestingly, an association between bleeding and AKI has also been specified previously. Since—in contrast to most widely used leaflet-based therapies—TTVA implies the use of contrast media for intraprocedural display of the right coronary artery, and the prevalence and implications of AKI are of great interest.

The incidence of renal failure is inconsistent in previous studies regarding patients undergoing tricuspid valve repair, ranging from 0 to 11.7% [6, 8, 20]. The reporting of renal endpoints was not standardized in the latter studies which makes data difficult to interpret and might cause underestimation of AKI. For instance, Davidson et al. reported in the early feasibility study an incidence of 0% after TTVA, as renal failure was defined solely as the necessity of new renal replacement therapy [20].

In the TRIVALVE registry, Taramasso et al. observed acute kidney injury in nine patients (3.6%) following transcatheter interventions to treat TR [21]. Disadvantageously, the exact definition of acute renal failure was not provided.

The recently published comparison between TTVA and transcatheter edge-to-edge repair (TEER) showed renal failure with new-onset replacement therapy in 4.6% (*n* = 4) in TEER and 3.1% (*n* = 1) in TTVA. So interestingly, there is no data showing a significantly higher amount of renal failure after TTVA, in spite of the periinterventional need for contrast media. Varying definitions of renal failure, on the other hand, limit the validity and comparability of data.

When defining AKI using the KDIGO criteria, the overall incidence of acute kidney injury in our study population was 18.6% (*n* = 27), accordingly, with two patients (1.4%) needing new renal replacement therapy.

The etiology of AKI after direct annuloplasty is probably multifactorial and may include contrast-induced nephropathy, atheroemboli, and other causes. Notably, in our study, there was no association of the amount of contrast media with the occurrence of AKI.

Another frequently discussed predictor of AKI is the duration of the procedure. A possible explanation seems to be intraprocedural hypotension, which has been linked to postoperative AKI in surgical mitral valve repair [10]. However, in our cohort, there was no significant difference in minimal periprocedural mean arterial pressure in patients with and without AKI (median 63 mmHg vs. 65 mmHg, *p* = 0.45). As mentioned before, the average procedure duration is longer in direct annuloplasty compared to leaflet approximation. The median procedure time of 198 min (up to 254 min in the TRI-REPAIR study) remains disadvantageously long, but was not significantly different in patients with AKI compared to patients without AKI.

In summary, the pathophysiology of postinterventional AKI seems to be multifactorial including the above as well as important cardiovascular risk factors such as impaired baseline renal function, diabetes mellitus, and arterial hypertension.

Importantly, in our study population, we did not see a significantly increased overall mortality at short-term follow-up in patients with acute kidney injury (7.4% vs. 8.5%, *p* = 0.83). This might be explained by the high percentage of mild/stage 1 acute kidney injury (24 out of 27) in our study population. Severe kidney injury (stage 3 according to MVARC criteria), which might be associated with worse patient outcome, occurred in only 3.4%.

### Study limitations

Several limitations must be considered. Inherent to the retrospective and observational study design are limitations like incompleteness of data. Nonetheless, we could obtain serial creatinine and Hb levels within the first 48 h for 94.5% (141/145) of patients. Furthermore, unmeasured confounders like changes in potential nephrotoxic or anticoagulation medication or medical history including specific kidney diseases might have an impact on bleeding and AKI not visible in our analysis. Also, urine output as part of the KDIGO AKI definition could not be measured retrospectively. Cohort size may limit statistical power to detect minor differences in effects; on the other hand, this is one of the largest multi-center real-world collectives of patients undergoing TTVA.

## Conclusions

The presented data suggest a relevant incidence of bleeding complications and AKI affecting about a fifth of patients undergoing transcatheter tricuspid valve annuloplasty (TTVA). None of these complications affected survival at short-term follow-up, but prolonged the hospital stay and the time spent on ICU.

The most frequent bleeding localizations were bleeding of the femoral access site, pericardial hemorrhage, and the esophagus, which need explicit attention in periprocedural management.

While TTVA is technically more complex than leaflet-based repair with longer procedure duration and need for arterial access and angiography of the right coronary artery, in this study neither procedure duration nor amount of contrast media was a predictor of AKI or bleeding.

### Impact on daily practice

This study determines the incidence and clinical impact of bleeding complications AKI following TTVA. While about a fifth of patients undergoing TTVA suffered from postinterventional AKI or bleeding, none of these complications was associated with higher mortality at short-term follow-up. One important risk factor for both complications was chronic renal dysfunction, indicating a high-risk patient population. The most frequent bleeding localizations were the femoral access site, pericardial hemorrhage, and the esophagus, which need explicit attention in periprocedural management. This knowledge may reduce risk of periinterventional complications.

## Data Availability

The data that support the findings of this study are available from the corresponding author, TG, upon reasonable request.

## Abbreviations

MVARC: Mitral Valve Academic Research Consortium
KDIGO: Kidney Disease: Improving Global Outcomes
TR: Tricuspid regurgitation
TEER: Tricuspid edge-to-edge repair
TTVA: Transcatheter tricuspid valve annuloplasty
AKI: Acute kidney injury
RBC: Red blood cell
RCA: Right coronary artery
BMI: Body mass index
ICU: Intensive care unit
